# Microstructural and Physical Properties of High‐Protein, High‐Overrun Frozen Desserts

**DOI:** 10.1111/1750-3841.70944

**Published:** 2026-02-26

**Authors:** Samantha R. VanWees, Scott A. Rankin, Richard W. Hartel

**Affiliations:** ^1^ University of Wisconsin‐Madison Madison Wisconsin USA

**Keywords:** frozen desserts, high protein, high overrun, microstructure, drip‐through, meltdown

## Abstract

Ice cream and frozen desserts fortified with protein often have undesirable physical and textural properties despite their increased nutritional value, and are susceptible to shrinkage during storage. The effects of dairy protein structure on structural and physical properties of the mix and frozen product were identified by studying frozen dessert mixes formulated to contain 6% milk protein concentrate, sodium caseinate, or whey protein isolate. The addition of 0.15% mono‐ and diglycerides decreased the mean ice crystal and air cell size in all frozen desserts and increased the degree of fat destabilization in frozen desserts made with milk protein concentrate and whey protein isolate, providing resistance to collapse during melting. The interfacial activity of serum proteins, casein micelles, nonmicellar casein, and mono‐ and diglycerides was essential to sequential formation and stabilization of structure, especially with regard to the emulsified fat globule membrane composition and formation of destabilized fat network during freezing. The correlation between rate of drip‐through during melting and degree of fat destabilization was independent of protein source, demonstrating how mix ingredient functionality influenced the co‐development of ice, air, fat, and serum phase structures in the frozen dessert, which ultimately governed the physical properties of the frozen dessert.

**Practical Applications:**

With the continued interest in high‐protein products, this work demonstrates the potential for selectively choosing type of protein to enhance physicochemical and microstructural properties of ice cream and frozen dairy desserts.

**Abbreviations**
MPC: milk protein concentrate
NaCN: sodium caseinate
WPI: whey protein isolate
MDG: mono‐ and diglycerides

## 1 Introduction

The term ‘frozen dessert’ is used to describe products similar to ice cream, gelato, sorbet, or similar products regardless of a federal standard of identity. The composition and processing of these products determine the final structure, which generally consists of ice crystals, air cells, fat globules, partially‐coalesced fat networks, and an unfrozen serum phase. In high‐overrun frozen desserts containing greater than 50% air by volume, the air phase must be stabilized by ingredients and the surrounding phases co‐developed during continuous freezing in order to maintain a quality product.

Protein ingredients are highly influential in developing the structure of the mix and frozen dessert due to their many functional properties. Immediately following homogenization, proteins stabilize the fat globule interface to prevent coalescence and flocculation during aging and increase the aqueous phase viscosity (Goff et al. 2025). Selective processing of dairy ingredients can concentrate or isolate specific dairy proteins, including casein micelles, nonmicellar casein, and serum or whey proteins, which offer unique functionality in aqueous and interfacial phases. Globular serum proteins denature when exposed to shear or heat stresses to expose hydrophobic regions and regions rich in sulfur‐containing amino acids to aid in formation and stabilization of fat or air phases. Casein proteins migrate freely to interfaces, and the phosphorylation and proline content in caseins yield disordered secondary and tertiary structures to stabilize dispersed systems. In native milk ingredients, caseins are assembled into highly porous micelles with additional functionality in emulsions and foams (Walstra et al. 2006).

Low molecular weight surfactants, such as mono‐ and diglycerides (MDG), are commonly added to frozen desserts due to their activity in fat phase development (Goff et al. 2025). During aging, these small molecules often displace some protein molecules from the interface to reduce interfacial tension, resulting in a mixed fat globule interface that facilitates partial coalescence of fat during dynamic freezing (Dickinson and Gelin 1992; Goff et al. 1987; Granger et al. 2005; Pelan et al. 1997). In mixes with no added MDG, the fat globule interface is stabilized primarily by proteins that provide steric resistance to coalescence during aging and freezing (Brooker 1993; Brooker et al. 1986; Goff et al. 1999).

The properties of the mix and frozen dessert are influenced by the structural components developed throughout the process, which in turn affect the melting behavior of the product at room temperature. In particular, increased fat destabilization (Bolliger et al. 2000; Goff and Jordan 1989; Segall and Goff 2002; Warren and Hartel 2018) or serum viscosity (Amador et al. 2017; Muse and Hartel 2004; Wu et al. 2019; Yuennan et al. 2014) has been shown to decrease the drip‐through rate and promote shape retention during melting. Other structural parameters such as small mean ice crystal and air cell size have been shown to correlate with reduced drip‐through rate, but the influence of structure varies widely depending on formulation and freezing processes (Wu et al. 2025).

The objective of this work was to gain insight as to how surface‐active ingredients and overrun influenced the ice, air, and fat structures of a high‐protein frozen dairy dessert, and the resulting impact on melting at room temperature.

## 2 Materials and Methods

Powdered milk protein concentrate (MPC), sodium caseinate (NaCN), or whey protein isolate (WPI) was used to formulate high‐protein frozen dessert mix with and without added MDG, then frozen to 100% or 150% overrun to investigate the structural and physical properties.

### 2.1 Materials

Frozen dessert mixes were prepared by combining anhydrous milk fat, lactose, sucrose, milk minerals, stabilizer, MDG, powdered dairy proteins, and water. Powdered MPC was obtained from Idaho Milk Products (Jerome, ID), NaCN was obtained from Milk Specialties Global (Eden Prairie, MN), and WPI was obtained from Glanbia Nutritionals (Twin Falls, ID). The composition of the powdered protein ingredients is shown in Table 1. The milk mineral blend was also obtained from Glanbia Nutritionals. Anhydrous milk fat (AMF) and crystalline lactose were obtained from Grassland Dairy (Greenwood, WI). Crystalline sucrose was obtained from Domino Foods (Yonkers, NY). MDG and the stabilizer blend of guar gum, locust bean gum, and carrageenan, were obtained from Danisco (New Century, KS).

**TABLE 1:** Composition (w/w%) of powdered milk protein concentrate (MPC), sodium caseinate (NaCN), and whey protein isolate (WPI).

Component	MPC	NaCN	WPI
Protein	80.40	90.34	91.01
Casein protein	64.32	90.34	—
Serum/whey protein	16.08	—	91.01
Fat	0.97	0.80	0.23
Ash	6.89	4.10	2.46
Lactose	6.59	0.40	3.29
Moisture	5.15	5.40	3.02

### 2.2 Methods

#### 2.2.1 Frozen Dessert Mix Production

Frozen dessert mixes were formulated to include 12.0% fat, 13.3% milk solids nonfat (6.0% protein, 6.3% lactose, and 1.0% ash), 14.5% sucrose, 0.2% stabilizer, and 0.0% or 0.15% MDG. The calculated freezing point (−2.60°C) was held constant for each batch. The quantities of AMF, powdered dairy protein (MPC, NaCN, WPI), lactose, milk minerals, and water were adjusted to achieve the formulation composition, while sucrose, stabilizer, and MDG (if present) were unchanged (Table 2).

**TABLE 2:** Composition of frozen dessert mixes manufactured with milk protein concentrate (MPC), sodium caseinate (NaCN), or whey protein isolate (WPI), with 0.0% or 0.15% added mono‐ and diglycerides (MDG).

Ingredient (%)	Mix formulation					
AMF	11.99	11.87	12.01	11.89	12.05	11.93
MPC powder	7.32	7.32	0	0	0	0
NaCN powder	0	0	6.54	6.54	0	0
WPI powder	0	0	0	0	6.48	6.48
Lactose	5.83	5.83	6.22	6.22	6.02	6.02
Milk minerals	0.58	0.58	0.86	0.86	1.00	1.00
Sucrose	14.51	14.51	14.51	14.51	14.51	14.51
MDG	0	0.15	0	0.15	0	0.15
Stabilizer	0.20	0.20	0.20	0.20	0.20	0.20
Water	59.58	59.55	59.66	59.63	59.74	59.71

Abbreviation: AMF = anhydrous milk fat.

Frozen dessert mixes were made using two separate heating processes to avoid excessive denaturation of protein during pasteurization. In the first process, powdered dairy protein was rehydrated with half of the liquid water in the formulation. The mixture was gently heated using a water bath (42°C) and stirred at low speed to avoid foaming for 60 min. Separately, melted AMF, sucrose, lactose, milk minerals, stabilizer, and MDG (if present) were combined with the remaining liquid water. The mixture was heated to 85°C using a jacketed mixer (Stephan Food Processing Machinery, Hamelin, Germany) to dissolve sugars and hydrate the hydrocolloid stabilizers, then cooled to 70°C.

The rehydrated protein mixture was added to the remaining ingredients, then homogenized using a two‐stage homogenizer (Manton‐Gaulin MFG Co. Inc., Everett, MA) set to 13.8 MPa on the first stage and 3.4 MPa on the second stage. The mix was returned to the jacketed mixer to cool to 20°C with gentle agitation, transferred to secondary containers, and aged overnight at 4°C. Two batches (22–25 kg each) for each formulation were manufactured and combined immediately prior to freezing. Each mix formulation was made in duplicate.

#### 2.2.2 Freezing and Hardening

A continuous freezer (Hoyer Frigus KF 80 F, Tetra Pak Hoyer Inc., Aarhus, Denmark) was used to manufacture all frozen desserts. Most conditions were maintained across all treatments, including dasher speed (500 RPM) and cylinder pressure (358.2 kPa). Air input was varied to achieve overrun of 100.0% (±0.43%) or 150.1% (±0.49%). Draw temperature varied slightly (−4.68 ± 0.33°C) to accommodate the high viscosity of mixes and facilitate aeration. The frozen desserts were packaged in 118.3 mL paper containers and immediately hardened at −29°C for at least 2 h, then stored at −29°C until analysis.

#### 2.2.3 Mix Analyses

##### 2.2.3.1 Apparent Viscosity

Mix viscosity was measured using a Discovery DHR‐2 hybrid rheometer (TA Instruments, Newcastle, DE) with a concentric cylinder geometry as described by Wu et al. (2019). A 23 mL aliquot of aged, homogenized mix was loaded in the temperature‐controllable cell and allowed to equilibrate to 0°C for 300 s prior to a flow sweep (100 to 1 s^−1^ shear rate). The resultant curve of stress as a function of shear rate was fitted using the Herschel–Bulkley model and the apparent viscosity at 50 s^−1^ obtained using TRIOS software (TRIOS Version 5.1.1, Newcastle, DE) was used to characterize the mixes. Flow sweeps were conducted within 48 h of mix manufacture and analyzed in triplicate.

##### 2.2.3.2 Density

Mix density was calculated as the ratio of mass to volume of a 5 mL aliquot of aged, homogenized mix. The average of five sample measurements at ambient temperature (21°C) was recorded as the mix density.

##### 2.2.3.3 Interfacial Tension

The air/liquid interfacial tension of the aged, homogenized mix was measured using a pendant drop method. In this method, a drop of liquid sample was suspended in the surrounding phase (air) and an optical method was used to calculate the interfacial tension was calculated using the Laplace equation. An 18 µL drop of mix was suspended in air at room temperature and a KRÜSS drop shape analyzer (DSA30R; KRÜSS Scientific, Hamburg, Germany) was used to measure the interfacial tension. Static equilibrium was achieved after 60 min for each mix and the equilibrium interfacial tension after was recorded in triplicate.

#### 2.2.4 Frozen Dessert Analyses

##### 2.2.4.1 Overrun

Product overrun describes the percentage increase in volume due to air incorporation during freezing. The mass of containers of fixed volume (103.5 mL) filled with mix or frozen dessert were measured periodically throughout the freezing process to ensure consistent output of frozen desserts. Overrun was calculated as the difference between mix mass and frozen product mass relative to the frozen product mass at equal volumes.

##### 2.2.4.2 Ice Crystal Size Distribution

Ice crystals were observed using the method of Donhowe et al. (1991). A refrigerated glovebox was set to −15°C, and an optical microscope (Accu‐scope 3000‐LED, Accu‐scope, Commack, NY) was used to take photos of ice crystals at 10× magnification. Prior to analysis, frozen desserts were tempered for 12 h at −20°C and then 30 min at −15°C immediately prior to observation. A sample of frozen dessert was taken from the center of the container using chilled tools and placed on a chilled microscope slide within the glovebox. One drop of chilled solvent (50% pentanol, 50% kerosene) was added to the sample to aid in dispersion of ice crystals before being covered by a chilled cover slip. Samples were spread to a single layer and approximately 20 images were captured using a Moticam 3+ microscope digital camera (Moticam, Kowloon, Hong Kong). At least 300 ice crystals were manually traced using Microsoft Paint and enumerated using Image Pro Plus (Version 7.0 Media Cybernetics 2009, Rockville, MD) to convert to enclosed area (µm^2^) and provide a representative sample size distribution. Each frozen dessert formulation was analyzed in duplicate and mean sizes of each distribution were averaged.

##### 2.2.4.3 Air Cell Size Distribution

Air cells were measured using the same refrigerated glovebox noted above according to the method of Chang and Hartel (2002a). Frozen desserts were tempered at −20°C for 12 h then 60–90 min at ‐15°C before observation. Specialized microscope slides were fabricated to create a well (125 µm deep) in which a sample of frozen dessert was placed then covered with a cover slip. The temperature of the glovebox was slowly raised to −6°C (1°C –2°C per min) to melt some ice crystals and promote visualization of air cells at 10× magnification. Approximately 10–15 images were captured using a microscope digital camera, and 300–400 circular air cells were traced and enumerated using Image Pro Plus for each sample. Each frozen dessert formulation was analyzed in duplicate and mean sizes of each distribution were averaged.

##### 2.2.4.4 Fat Particle Size Distribution

The particle size distributions of emulsified fat globules in frozen dessert mix and fat globule clusters in melted frozen desserts were measured using laser light scattering (Malvern Mastersizer 3000, Malvern Instruments Ltd., Worcestershire, UK). Mixes and melted frozen desserts were stored at 4°C for no more than 24 h prior to analysis, then liquid samples were added dropwise until the laser obscuration value reached 13%–15%. Deionized water was used as a dispersant (refractive index of 1.33). The refractive index of milkfat was set to 1.47 and absorbance was set to 0.01. Six measurements were collected per sample aliquot, and two aliquots were measured for each mix formulation.

Two distinct peaks were visible in melted frozen desserts: one representing individual milkfat globules (0.6–1.2 µm) and one representing clusters of destabilized fat (10–100 µm) (Goff et al. 2025). The percentage of destabilized fat was calculated as the ratio of the volume percentage of destabilized fat in the melted frozen dessert compared to the volume percentage of fat present in the mix. To visually confirm the presence of destabilized fat, three drops of sample were diluted with 20 mL deionized water then viewed using optical microscopy (Nikon Labophot‐2 microscope, Tokyo, Japan). The diluted sample was placed on a chilled glass microscope slide and covered with a cover slip. Samples were observed at 40× magnification.

##### 2.2.4.5 Melting Behavior

Melting behavior was measured using a drip‐through test modeled after the method described by Bolliger et al. (2000). Frozen desserts were removed from storage and tempered for 12 h at −20°C prior to analysis. The entire 103.5 mL container was used for the meltdown evaluation. Packaging was removed and the weight of the frozen dessert (approximately 4.0 mm in height and 7.4 mm in diameter) was recorded before the slab was placed on a wire mesh screen (three openings per cm). The mesh and frozen dessert were positioned 20 cm above a beaker to collect drops, and allowed to melt at ambient temperature (22°C) for 6 h or until melting reached a plateau. The mass of sample that had dripped through the mesh was recorded automatically every 60 s for 360 min. The relative quantity (%) of frozen dessert that had dripped through the mesh was plotted as a function of time and the linear portion was recorded as the drip‐through rate. Each formulation was analyzed in triplicate.

#### 2.2.5 Statistics

Statistical analysis was conducted using statistical software (R Version 3.3, Posit, Boston, MA). One‐way ANOVA tests with Tukey's HSD post‐hoc analyses (*α* = 0.05) were conducted to determine the effects of protein source and MDG addition on mix responses, and to determine effects of protein source, MDG addition, and overrun on responses in frozen desserts prior to storage. Linear regression analysis was used to determine correlations between variables.

## 3 Results and Discussion

### 3.1 Mix Properties

There were no significant effects of protein source or MDG addition on density of the frozen dessert mixes (Table 3), and mix density was within the expected standard range for frozen dessert mixes of 12% fat and 40% solids (Goff et al. 2025).

**TABLE 3:** Properties of frozen dessert mixes made with 6% milk protein concentrate (MPC), sodium caseinate (NaCN), or whey protein isolate (WPI) and varying levels of mono‐ and diglycerides (MDG).

Protein source	MDG (%)	Density (g mL^−1^)	Interfacial tension (mN m^−1^)	Viscosity (mPa·s)
MPC	0.0	1.11 ± 0.00 ^a, A^	45.6 ± 1.36 ^a, A^	299 ± 7.30 ^a, A^
	0.15	1.12 ± 0.00 ^a, A^	41.8 ± 0.35 ^a, B^	359 ± 19.4 ^a, B^
NaCN	0.0	1.12 ± 0.01 ^a, A^	46.7 ± 0.28 ^a, A^	466 ± 22.2 ^b, A^
	0.15	1.12 ± 0.01 ^a, A^	41.3 ± 0.31 ^ab, B^	507 ± 30.5 ^b, A^
WPI	0.0	1.11 ± 0.01 ^a, A^	44.8 ± 1.36 ^a, A^	123 ± 0.57 ^c, A^
	0.15	1.13 ± 0.01 ^a, A^	39.2 ± 0.26 ^b, B^	134 ± 3.90 ^c, A^

*Note*: Mean ± SD are shown. Superscript (a, b, c) denote significant differences by protein source. Superscript (A, B) denote significant differences by MDG addition. Values in the same column sharing a letter are not significantly different at *α* = 0.05.

All mixes displayed an interfacial tension lower than that of a pure air/water interface (72 mN m^−1^) after 60 min of equilibration, either with or without MDG. As expected, mixes made with 0.15% MDG had significantly lower interfacial tension for all protein sources due to the migration of MDG to the newly‐formed interface. Mixes made with WPI had the lowest interfacial tension while mixes made with NaCN had the highest; however, these differences with protein source were only significant when 0.15% MDG were added.

Mixes made with WPI had the lowest apparent viscosity, whereas mixes made with NaCN had the highest. The addition of 0.15% MDG increased the apparent viscosity for all protein sources, but this increase was only significant in mixes made with MPC. The addition of MDG was not expected to affect the rheological properties of the bulk emulsion; therefore, the effects of MDG on apparent viscosity were related to the development of the milkfat globule interface, where competitive displacement of protein by MDG during aging increased the concentration of protein in the aqueous phase and resulted in a more viscous mix.

The hydration, size, and functionality of caseins in dispersions and emulsions has been shown to increase the apparent viscosity of aqueous dispersions (Pitkowski et al. 2008) and ice cream mixes (Huppertz et al. 2011). Mixes made with MPC contained some nonmicellar casein, but the concentration was not sufficient to affect the apparent viscosity, and undenatured serum proteins had limited functionality in the mixes made with WPI (Britten and Giroux 1991a).

### 3.2 Structural Properties of Frozen Desserts

#### 3.2.1 Ice Crystal Size and Distribution

Mean ice crystal size ranged from 28.7 to 41.9 µm (Table 4), in good agreement with typical ice crystal size in frozen desserts (Goff et al. 2025). Overall, the shape of the size distributions and morphology of ice crystals were unaffected by protein source, MDG addition, or overrun (Table 5). Frozen desserts made with NaCN had significantly higher mean ice crystal size compared to MPC and WPI, which was likely due to the difference in draw temperature for mixes made with NaCN rather than the protein source. Frozen dessert mixes made with MPC and WPI were frozen to −4.86°C (±0.05°C) and −4.92°C (±0.14°C), respectively, but mixes made with NaCN were frozen to −4.26°C (±0.03°C) to prevent over freezing. While this increase in draw temperature was not excessive, research has shown that draw temperature affects ice phase volume and mean ice crystal size following hardening (Amador et al. 2017; Inoue et al. 2012).

**TABLE 4:** Structural properties of frozen desserts made with 6% milk protein concentrate (MPC), sodium caseinate (NaCN), or whey protein isolate (WPI) and varying levels of mono‐ and diglycerides (MDG) and overrun.

Protein source	MDG (%)	OR (%)	Mean ice crystal size (µm)	Mean air cell size (µm)	Fat destabilization (%)
MPC	0.0	100	35.7 ± 0.54 ^a, A, x^	35.0 ± 0.55 ^a, A, x^	4.73 ± 1.18 ^a, A, x^
		150	31.3 ± 0.49 ^a, A, y^	32.3 ± 0.45 ^a, A, y^	4.26 ± 0.31 ^a, A, x^
	0.15	100	32.8 ± 0.03 ^a, B, x^	28.9 ± 0.53 ^a, B, x^	19.4 ± 5.08 ^a, B, x^
		150	30.2 ± 0.09 ^a, A, y^	25.1 ± 0.03 ^a, B, y^	26.7 ± 3.49 ^a, B, y^
NaCN	0.0	100	41.9 ± 0.49 ^b, A, x^	35.4 ± 0.74 ^a, A, x^	3.53 ± 0.51 ^a, A, x^
		150	37.6 ± 0.72 ^b, A, y^	36.7 ± 0.28 ^b, A, x^	3.82 ± 0.70 ^a, A, x^
	0.15	100	40.3 ± 0.23 ^b, B, x^	36.8 ± 2.12 ^b, A, x^	3.87 ± 0.31 ^b, A, x^
		150	36.5 ± 0.93 ^b, A, y^	35.3 ± 0.58 ^b, A, x^	3.71 ± 1.99 ^b, A, x^
WPI	0.0	100	32.5 ± 0.73 ^c, A, x^	34.0 ± 1.35 ^a, A, x^	7.22 ± 0.16 ^a, A, x^
		150	30.1 ± 0.46 ^a, A, y^	31.2 ± 0.19 ^a, A, y^	8.66 ± 0.40 ^a, A, x^
	0.15	100	33.7 ± 0.81 ^a, A, x^	31.1 ± 0.16 ^a, B, x^	34.8 ± 1.09 ^c, B, x^
		150	28.7 ± 0.20 ^c, B, y^	27.2 ± 0.33 ^a, B, y^	45.2 ± 1.19 ^c, B, x^

*Note*: Mean ± SD are shown. Superscript (a, b, c) denote significant differences with protein source, superscript (A, B) denote significant differences by MDG addition, superscript (x, y) denote significant differences by overrun. Values in the same column sharing a letter are not significantly different at *α* = 0.05.

**TABLE 5:** Optical micrographs of ice crystals in frozen desserts made with 6% milk protein concentrate (MPC), sodium caseinate (NaCN), or whey protein isolate (WPI), and varying levels of mono‐ and diglycerides (MDG) and overrun (OR). Scale bar = 200 µm.

MDG (%)	OR (%)	Protein source		
		MPC	NaCN	WPI
0.0	100	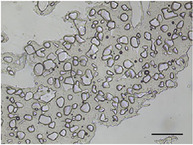	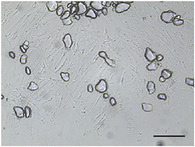	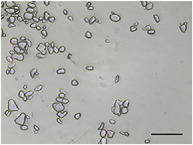
	150	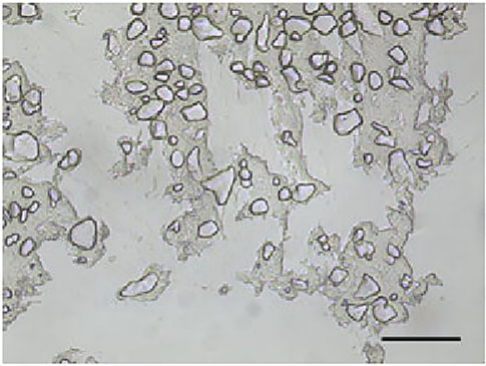	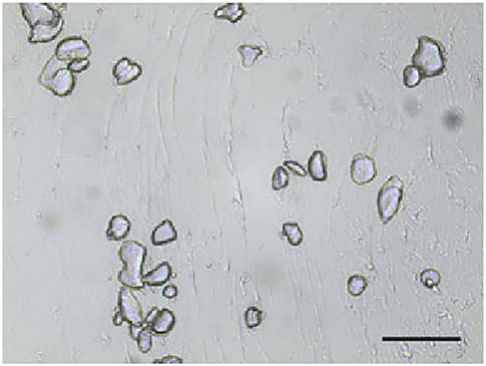	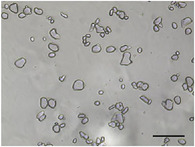
0.15	100	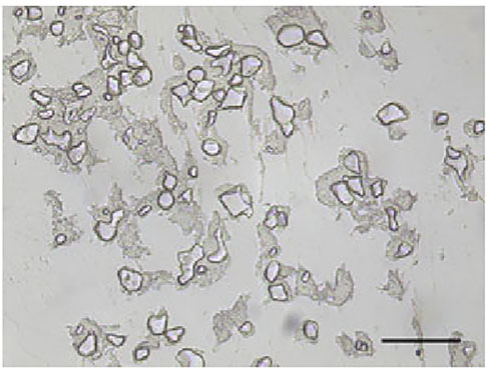	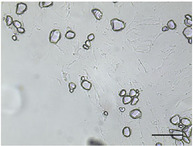	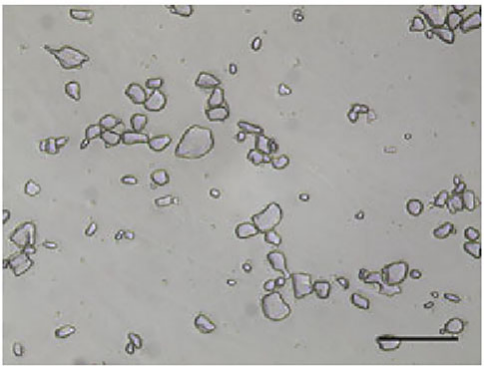
	150	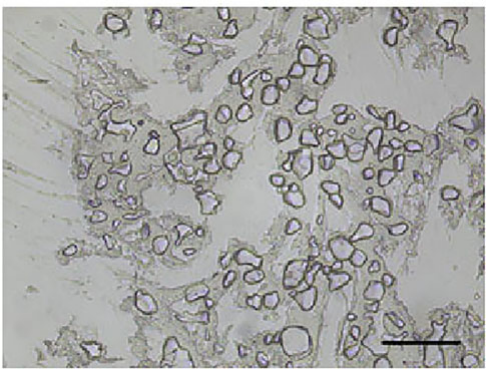	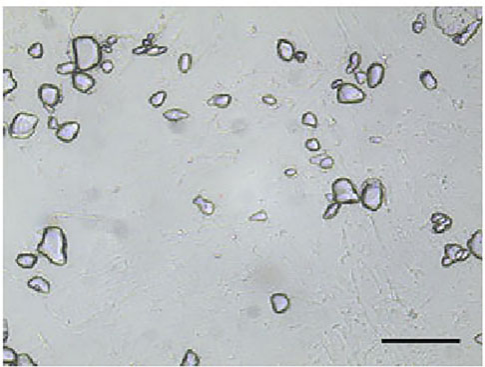	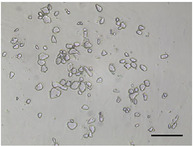

The mean ice crystal size decreased for all frozen desserts as overrun increased. This was attributed to the restricted mobility of water caused by high solute concentration in the thin lamellar phase in the high‐overrun products, as previously shown by (Caillet et al. 2003; Flores and Goff 1999; Goff 1997a; Hernández Parra et al. 2018; Sofjan and Hartel 2004). In addition, locally concentrated regions of polysaccharides and/or proteins and destabilized fat networks may have further reduced the ability for ice crystals to grow during hardening. Frozen desserts made with the same protein source and 0.15% MDG had slightly lower mean ice crystal size compared to those made with 0.0% MDG, but the effects were not significant nor consistent.

#### 3.2.2 Air Cell Size and Distribution

The mean air cell size ranged from 25.1 to 36.8 µm (Table 4), within the range expected for frozen desserts with similar composition (Goff et al. 2025). Continuous freezing resulted in uniform air cell size distributions and morphology for frozen desserts across all protein sources, MDG addition levels, and overrun (Table 6).

**TABLE 6:** Optical micrographs of air cells in frozen desserts made with 6% milk protein concentrate (MPC), sodium caseinate (NaCN), or whey protein isolate (WPI), and varying levels of mono‐ and diglycerides (MDG) and overrun (OR). Scale bar = 200 µm.

MDG (%)	OR (%)	Protein source		
		MPC	NaCN	WPI
0.0	100	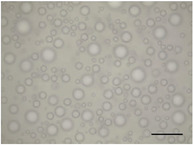	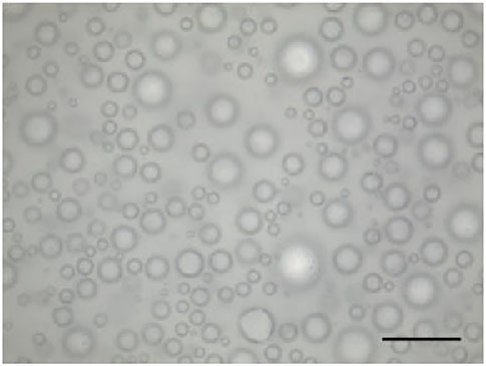	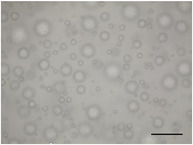
	150	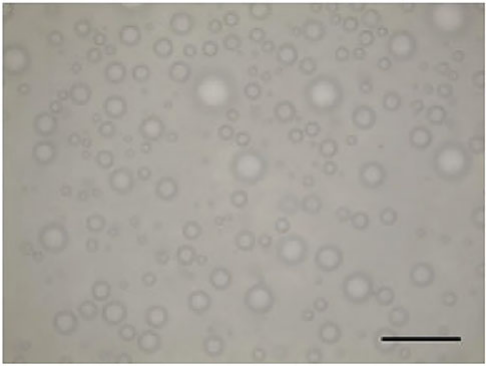	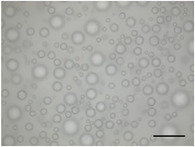	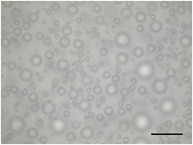
0.15	100	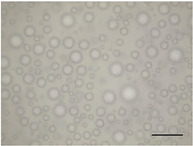	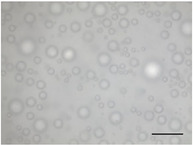	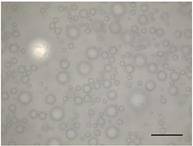
	150	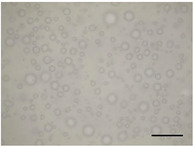	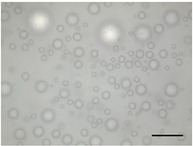	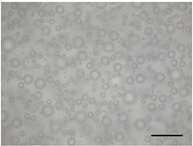

Frozen desserts made with NaCN had significantly higher mean air cell size compared to those made with MPC or WPI. The addition of 0.15% MDG and increase in overrun also reduced the mean air cell size in frozen desserts made with MPC and WPI, but had no effects on those made with NaCN, suggesting that the nonmicellar casein had unique interfacial functionality. The higher mean air cell size may also have been attributed to the higher draw temperature (Caillet et al. 2003; Eisner et al. 2005; Hernández Parra et al. 2018; Inoue et al. 2008; Rohenkohl and Kohlus 1999), or increased mix viscosity (Goff et al. 1989); however, the difference in air cell size was more likely related to the interfacial activity of caseins.

As mentioned previously, the aeration of frozen desserts is highly complex. Air cells are initially stabilized by proteins, and foamability of proteins is governed by the ability to migrate to and stabilize the interface (Damodaran 1994; Goff et al. 1999). During the initial stages of freezing, flexible proteins may migrate freely to the interface, and globular proteins form a tightly packed, viscoelastic interface (Carrera Sánchez and Rodríguez Patino 2005). As the dasher continues to disrupt the air phase, interfacial protein prevents coalescence and enables the formation of discrete air cells throughout the frozen dessert. Though the measured air/mix interfacial tension was highest in mixes made with NaCN, the small size and flexibility of caseins facilitated the incorporation of air compared to micellar casein or globular serum proteins (Damodaran 1994; Martínez‐Padilla et al. 2014).

In frozen desserts made with MPC and WPI, the air cell size decreased with addition of MDG and at higher overrun. This may have been due to the increase in apparent viscosity of the mixes made with 0.15% MDG, which has been correlated with lower mean air cell size at draw (Chang and Hartel 2002b; Eisner et al. 2005) and decreased growth during hardening (Goff et al. 1999). The overall air/serum interfacial area increased as air cells were broken down during freezing, and the higher concentration of aqueous protein in mixes made with 0.15% MDG may have prevented coalescence in the freezer and during hardening.

Researchers have also shown mean air cell size to decrease with increasing overrun (Sofjan and Hartel 2004; Warren and Hartel 2018); however, this was not observed in frozen desserts made with NaCN. Since the protein concentration was above the concentration needed to form a monolayer at the oil and air interfaces, the casein‐stabilized interfaces were uniquely resistant to shear destabilization during dynamic freezing.

#### 3.2.3 Fat Globule Size Distribution

**FIGURE 1:**
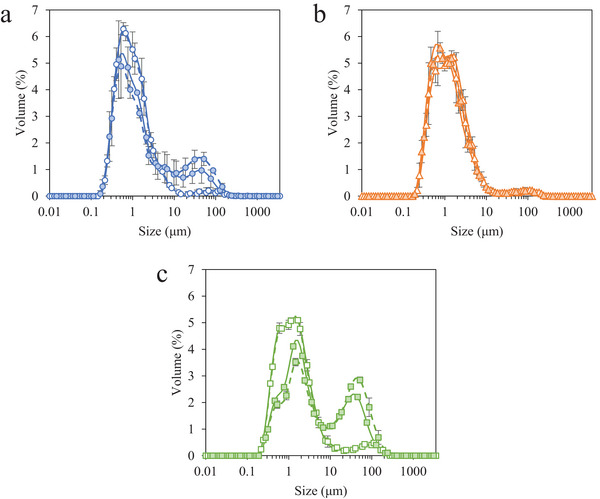
Fat globule size distributions for frozen desserts made with 6% milk protein concentrate (a), sodium caseinate (b), or whey protein isolate (c). Mono‐ and diglyceride addition of 0.0% (open symbols) or 0.15% (filled symbols) and overrun of 100% (solid lines) or 150% (dashed lines) are shown. Error bars represent the standard deviation of mean values measured in duplicate.

The addition of 0.15% MDG significantly increased the degree of fat destabilization, depicted as a peak in the range of 30–100 µm in the particle size distributions (Figure 1), in frozen desserts made with MPC and WPI but had no effect on those made with NaCN (Table 4). The competitive displacement of proteins by MDG results in a thinner milkfat globule interface that is more susceptible to shear‐induced partial coalescence (Barfod 2001; Bolliger et al. 2000; Goff and Jordan 1989). The saturated MDG in the added blend effectively displaced micellar and nonmicellar caseins from fat interfaces (Cheng et al. 2020; Davies et al. 2000; Munk et al. 2014), and the unsaturated MDG enhanced fat destabilization in aerated emulsions (Barfod 2001; Goff and Jordan 1989; Lee et al. 2018), providing optimal conditions for the frozen desserts studied. In mixes with 0.0% MDG, the high surface concentration of proteins and the steric hindrance of interfacial protein molecules likely prevented fat destabilization (Dalgleish 1996; Oortwijn and Walstra 1979; Segall and Goff 1999).

Casein films have been shown to resist partial coalescence (Britten and Giroux 1991b; Goff et al. 1987; Pelan et al. 1997; Segall and Goff 1999), which may explain why very little fat destabilization was observed in frozen desserts made with NaCN. In the casein‐stabilized emulsions, MDG adsorbed to small imperfections in the protein layer to reduce the interfacial tension without displacing protein from the interface, maintaining a high surface concentration of interfacial protein (Cheng et al. 2020; Munk et al. 2014). The strong, cohesive interface prevented the coalescence of fat globules in all frozen desserts made with NaCN. Additional mechanisms of stabilization may include the complexation of caseins with MDG (Jiang et al. 2018; Munk et al. 2014) or pickering stabilization of saturated monoglycerides (Barfod et al. 1991; Goibier et al. 2017) at the fat globule interface.

When 0.15% MDG were added to mixes made with MPC, casein micelles and serum proteins were displaced from the interface, reducing the interfacial tension and promoting fat destabilization during freezing (Zhang and Goff 2005). The displacement of serum proteins by MDG is favorable compared to caseins (Mackie et al. 2001), further weakening the interface to shear‐induced destabilization, resulting in the highest degree of fat destabilization in frozen desserts made with WPI and 0.15% MDG. Globular serum proteins form a thick, elastic interfacial layer, but shear‐induced disruption of this rigid film increases the susceptibility to destabilization in emulsions made with WPI compared to those made with MPC or NaCN (Daw and Hartel 2015; Goff 1997b; Goff et al. 1989; Segall and Goff 1999).

The extent of fat destabilization was generally unaffected by overrun, especially when protein‐stabilized interfaces were more stable to shear‐induced partial coalescence, as in the case of frozen desserts made with 0.0% MDG or NaCN. When air phase volume increased in frozen desserts containing 0.15% MDG and MPC or WPI, the relative influence of internal and interfacial forces that govern shear‐induced partial coalescence were favorable to destabilization due to the weakening of protein domains by MDG (Fuller et al. 2015; Thiel et al. 2016).

### 3.3 Melting Behavior of Frozen Desserts

The collapse of the foam structure at ambient temperatures was quantified by studying the melting and drip‐through behavior of frozen desserts over time. Semisolid structure provided by the fat, air, and diluted serum phases, and physical properties such as interfacial tension and apparent viscosity influenced the time for the frozen dessert to begin dripping (induction time), the drip‐through rate, and the shape retention of the melted product.

**FIGURE 2:**
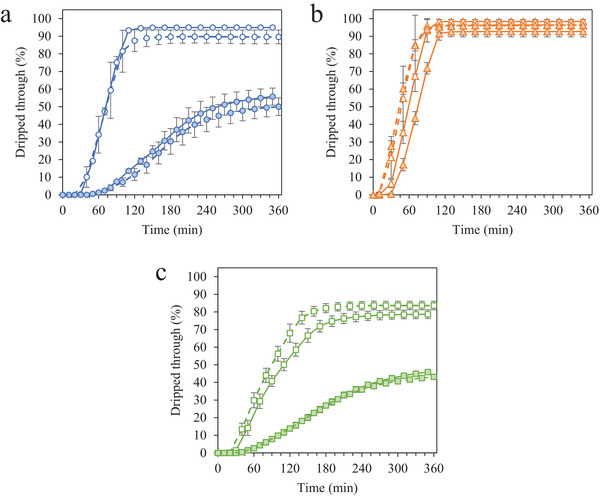
Drip‐through behavior of frozen desserts made with 6% milk protein concentrate (a), sodium caseinate (b), or whey protein isolate (c). Mono‐ and diglyceride addition of 0.0% (open symbols) or 0.15% (filled symbols) and overrun of 100% (solid lines) or 150% (dashed lines) are shown. Error bars represent the standard deviation of mean values measured in duplicate.

All frozen desserts displayed characteristic sigmoidal curves for drip‐through as a function of time (Figure 2). The effects of MDG addition and overrun on induction time, drip‐through rate, and shape retention were visible in frozen desserts made with MPC and WPI, whereas all frozen desserts made with NaCN showed similar melting behavior (Table 7). Further details regarding the melting behavior of the frozen desserts can be found in VanWees (2024).

**TABLE 7:** Melting properties of frozen desserts made with 6% milk protein concentrate (MPC), sodium caseinate (NaCN), or whey protein isolate (WPI) and varying levels of mono‐ and diglycerides (MDG) and overrun.

Protein source	MDG (%)	OR (%)	Induction time (min)	Drip‐through rate (% min^−1^)	Total dripped through (%)
MPC	0.0	100	27.9 ± 1.92 ^a, A, x^	1.39 ± 0.09 ^a, A, x^	95.0 ± 0.61 ^a, A, x^
		150	26.0 ± 3.27 ^a, A, y^	1.32 ± 0.21 ^a, A, y^	89.6 ± 3.92 ^a, A, x^
	0.15	100	42.8 ± 0.34 ^a, B, x^	0.311 ± 0.08 ^a, B, x^	56.0 ± 4.78 ^a, B, x^
		150	46.9 ± 5.15 ^a, B, y^	0.336 ± 0.07 ^a, B, y^	50.1 ± 5.03 ^a, B, y^
NaCN	0.0	100	22.9 ± 1.19 ^b, A, x^	1.61 ± 0.14 ^a, A, x^	98.4 ± 0.99 ^a, A, x^
		150	21.5 ± 5.18 ^b, A, y^	1.74 ± 0.23 ^b, A, x^	96.1 ± 3.39 ^a, A, x^
	0.15	100	30.5 ± 0.81 ^b, B, x^	1.39 ± 0.04 ^b, A, x^	92.7 ± 3.06 ^b, A, x^
		150	18.1 ± 1.98 ^b, A, y^	1.54 ± 0.05 ^b, A, x^	96.3 ± 1.62 ^b, A, x^
WPI	0.0	100	24.6 ± 0.92 ^c, A, x^	0.769 ± 0.05 ^a, A, x^	78.7 ± 2.47 ^a, A, x^
		150	21.1 ± 2.11 ^a, A, y^	0.792 ± 0.04 ^a, A, y^	83.7 ± 2.41 ^a, A, x^
	0.15	100	36.8 ± 3.79 ^a, A, x^	0.223 ± 0.00 ^a, B, x^	46.4 ± 0.30 ^c, B, x^
		150	36.3 ± 0.87 ^c, B, y^	0.218 ± 0.00 ^a, B, y^	43.2 ± 1.55 ^c, B, y^

*Note*: Mean ± SD are shown. Superscript (a, b, c) denote significant differences by protein source. Superscript (A, B) denote significant differences by MDG addition. Superscript ^(^x, y) denote significant differences by overrun. Values sharing a letter are not significantly different at *α* = 0.05.

The time for frozen desserts to begin dripping ranged from 18.1 to 46.9 min (Table 7). Of the three proteins sources, frozen desserts made with NaCN had the lowest induction times; and, in general, increasing overrun decreased the induction time, except for those with high levels of fat destabilization. The addition of MDG significantly increased the induction time for frozen desserts made with MPC and WPI at both 100% and 150% overrun but had little effect on frozen desserts made with NaCN.

Many factors affect the induction time, which is ultimately determined by the ability of a drop to form and fall from the screen. The air/mix interfacial tension did not affect the induction time (*r* = −0.571), suggesting that a combination of structural, thermal, and interfacial properties affected the drop formation and release. Previous researchers have shown that mix apparent viscosity positively correlated with induction time and independent of air phase volume and fat destabilization (Wu et al. 2019); however, this trend was not observed in this study. The presence of destabilized fat delayed the induction time in the frozen desserts studied by preventing liquid drainage and reducing the thermal diffusivity of the surface. Similarly, induction time decreased with overrun for most frozen desserts due to the insulating effects of air that prevented the flow of liquid and the reduced ice phase volume in a sample volume, as shown by Wu (2023).

The drip‐through rate was strongly affected by the structure and formulation of the frozen desserts (Table 7). Frozen desserts made with NaCN had the highest drip‐through rate, which was independent of MDG addition and overrun. For frozen desserts made with MPC and WPI, the drip‐through rate decreased with increasing overrun, in agreement with previous studies (Sakurai et al. 1996; Sofjan and Hartel 2004; Warren and Hartel 2018), and increased significantly with the addition of 0.15% MDG.

The effect of MDG on drip‐through rate in frozen desserts has been attributed largely to the presence of destabilized networks (Baer et al. 1997; Bolliger et al. 2000; Cropper et al. 2013; Koxholt et al. 2001). Destabilized fat networks provided structure to the foam by stabilizing air cells and restricting the flow of liquid through foam lamellae in frozen desserts made with MPC and WPI and 0.15% MDG (Koxholt et al. 2001; Muse and Hartel 2004). Frozen desserts made with WPI and 0.0% MDG had some fat destabilization, but the reduction in the drip‐through rate in these samples was more likely influenced by the properties of serum proteins. Previous researchers have also found that WPI reduced drainage and collapse in foams (Marinova et al. 2009; Tamm et al. 2012) and in melting ice cream (Daw and Hartel 2015). This may have been due to the interfacial adsorption of WPI, which prevented the flow of liquid through the lamella.

Despite the presence of serum proteins in frozen desserts made with MPC, a high drip‐through rate was observed in those made with 0.0% MDG. The mixed interfacial systems of caseins, serum proteins, and micellar casein likely contributed to the formation of a stable film at the fat globule interface, reducing the susceptibility to fat destabilization, and the air interface (Damodaran and Sengupta 2003; Gaiani et al. 2011; Zhang and Goff 2004). In addition, the high drip‐through rate in this system may have been due to the competitive displacement of serum proteins by caseins and casein micelles, which has been shown to reduce foam stability (Anand and Damodaran 1996).

Like drip‐through rate, the amount of frozen dessert remaining on the screen was affected by the extent of fat destabilization (Table 7). Frozen desserts made with NaCN had low fat destabilization and melted almost completely through the screen, as did samples made with MPC and 0.0% MDG. Frozen desserts made with WPI and 0.0% MDG had some remnant foam, and those made with MPC and WPI in the presence of 0.15% MDG had significantly more shape retention following 6 h of melting.

Frozen desserts with higher mean ice crystal size and mean air cell size had greater total drip‐through (*r* = 0.659, *r* = 0.864, respectively), and fat destabilization had the strongest effect on shape retention (*r* = −0.943). Destabilized fat networks reduced the flow of liquid and provided a scaffold structure to entrap air, fat, and aqueous phases atop the screen (Koxholt et al. 2001; Muse and Hartel 2004; Wu et al. 2019). Frozen desserts with 150% overrun showed greater shape retention due to the presence of many small air cells and high fat destabilization.

### 3.4 Effects of Physicochemical Parameters on Melting Properties

In this study, there were only marginal effects of ice crystal size or air cell size on any melting properties, and the air/mix interfacial tension and apparent mix viscosity had slight effects. The extent of fat destabilization was the only structural component that significantly affected the melting properties.

Recent studies have investigated the influence of interfacial tension on melting parameters, as the nature of how droplets forming under the screen has some effect on dripping behavior. For example, Hung and Yao (2002) proposed an equation to model the size of a drop (*D*′) falling from a wire mesh screen (Equation 1).

(1) $$D^{\prime }={\left[\frac{12\sigma }{\pi \rho_{L}g}D\right]}^{1/3}$$

In this equation, *σ* represents the interfacial tension, *ρ_L_* represents the liquid density, and *D* represents the size of the mesh screen openings. Wu (2023) demonstrated the validity of this equation in aerated sorbet systems, where samples with higher air/mix interfacial tension resulted in a larger droplet size and more rapid drip‐through rate. A correlation between air/mix interfacial tension and drip‐through rate was also observed in this study (*r* = 0.668).

**FIGURE 3:**
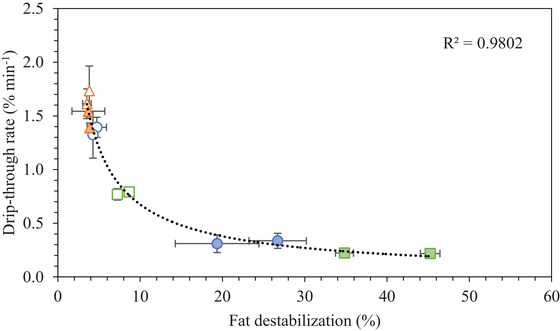
Drip‐through rate plotted as a function of fat destabilization for frozen desserts made with 6% milk protein concentrate (°,●), sodium caseinate (△,▲), or whey protein isolate (◻,◼). Mono‐ and diglyceride addition of 0.0% (open symbols) or 0.15% (filled symbols) are shown.

There was a significant correlation between drip‐through rate and the extent of fat destabilization, shown in Figure 3. The exponential relationship is given in Equation (2), where DT rate represents the drip‐through rate and FD represents the percentage of fat destabilization.

(2) $$\mathrm{DT}\;\mathrm{rate}\;=4.60{\left(\mathrm{FD}\right)}^{-0.834}$$

Numerous studies have correlated drip‐through rate with extent of fat destabilization, although with mixed results (Bolliger et al. 2000; Goff and Jordan 1989; Koxholt et al. 2001; Muse and Hartel 2004; Segall and Goff 2002; Warren and Hartel 2018; Wu et al. 2019). To date, this study has demonstrated the strongest correlation. The materials used in this study were selected to achieve a range of interfacial and physicochemical functionality to evaluate the effects on frozen dessert structural and physical properties. As a result, a range of ice, air, and fat structural sizes were achieved in the frozen desserts, elucidating a strong correlation between drip‐through rate and fat destabilization.

## 4 Conclusion

The type of dairy protein, along with the interaction of proteins and MDG at interfaces and in the bulk, affected the properties of the mix emulsion and the development of microstructure in high‐protein, high‐overrun frozen desserts. The displacement of proteins by MDG from the fat globule interface during aging affected the apparent viscosity and interfacial tension of the mix, and the size distributions of ice, air, and fat in the frozen dessert, which affected the melting properties. Globular serum proteins and micellar casein were readily displaced by MDG, which facilitated fat destabilization during freezing, whereas flexible caseins were not displaced. The scaffold‐like structure of destabilized fat delayed the onset of liquid dripping, reduced the drip‐through rate, and increased the shape retention during melting.

This study identified a strong exponential correlation between fat destabilization and drip‐through rate, indicating the importance of fat globule structures on melting rate. Further, evidence of the complexity of interfacial and bulk properties and their effects on the structural development of mixes and frozen desserts was demonstrated.

## Author Contributions

**Samantha R. Vanwees**: conceptualization, investigation, writing – original draft, methodology, visualization, writing – review and editing, formal analysis, data curation. **Scott A. Rankin**: funding acquisition, writing – review and editing, project administration, supervision, conceptualization, validation. **Richard W. Hartel**: project administration, supervision, writing – review and editing, funding acquisition, conceptualization, validation.

## Conflicts of Interest

The authors declare no conflicts of interest.

## Data Availability

Data are available upon reasonable request.
